# Construction and Application of an Electronic Spatiotemporal Expression Profile and Gene Ontology Analysis Platform Based on the EST Database of the Silkworm, *Bombyx mori*

**DOI:** 10.1673/031.010.11401

**Published:** 2010-07-23

**Authors:** Li- Ping Gan, Wen-Yu Zhang, Yan-Shan Niu, Li Xu, Jian Xi, Ming-Ming Ji, Shi-Qing Xu

**Affiliations:** ^1^ National Engineering Laboratory for Modem Silk, Department of Applied Biology, Medical College of Soochow University, Suzhou, 215153, P. R. China; ^2^ Biology Department, Chongqing Three Gorges University, Chongqing, 404000, China; ^3^ Bioinformatics Department, Medical College, Soochow University, Suzhou, 215153, China

**Keywords:** EST analysis package, UniGene, Lepidoptera

## Abstract

An Expressed Sequence Tag (EST) is a short sub-sequence of a transcribed cDNA sequence. ESTs represent gene expression and give good clues for gene expression analysis. Based on EST data obtained from NCBI, an EST analysis package was developed (apEST). This tool was programmed for electronic expression, protein annotation and Gene Ontology (GO) category analysis in *Bombyx mori* (L.) (Lepidoptera: Bombycidae). A total of 245,761 ESTs (as of 01 July 2009) were searched and downloaded in FASTA format, from which information for tissue type, development stage, sex and strain were extracted, classified and summed by running apEST. Then, corresponding distribution profiles were formed after redundant parts had been removed. Gene expression profiles for one tissue of different developmental stages and from one development stage of the different tissues were attained. A housekeeping gene and tissue-and-stage-specific genes were selected by running apEST, contrasting with two other online analysis approaches, microarray-based gene expression profile on SilkDB (BmMDB) and EST profile on NCBI. A spatio-temporal expression profile of *catalase* run by apEST was then presented as a three-dimensional graph for the intuitive visualization of patterns. A total of 37 query genes confirmed from microarray data and RT—PCR experiments were selected as queries to test apEST. The results had great conformity among three approaches. Nevertheless, there were minor differences between apEST and BmMDB because of the unique items investigated. Therefore, complementary analysis was proposed. Application of apEST also led to the acquisition of corresponding protein annotations for EST datasets and eventually for their functions. The results were presented according to statistical information on protein annotation and Gene Ontology (GO) category. These all verified the reliability of apEST and the operability of this platform. The apEST can also be applied in other species by modifying some parameters and serves as a model for gene expression study for Lepidoptera.

## Introduction

An Expressed Sequence Tag (EST) is a short sub-sequence of a transcribed cDNA sequence and can be considered to represent one gene (Adams et al. 1991). EST abundance can be taken as an approximation of the amount of gene expression in a given sample, so EST data could serve as a good resource for gene expression analysis (Ewing et al. 1999). Statistical analysis of the number of ESTs associated with specific cDNA libraries has allowed the calculation of probabilities of differential expression between different tissues (Chen et al. 2006). The EST database (dbEST) (http://www.ncbi.nlm.nih.gov/dbEST) is based on rough EST sequences with a high degree of redundancy and many errors, which need to be clustered and spliced. Therefore, a secondary database named UniGene (http://www.ncbi.nlm.nih.gov/UniGene) containing ESTs of the corresponding genes was generated (Zhuo et al. 2001; Pontius et al. 2003) and used to acquire all ESTs of each gene. On NCBI, the EST profile of UniGene shows breakdowns by body sites and developmental stage, but it cannot present them simultaneously. Therefore, it is necessary to develop a spatio-temporal expression mode enabling the application of a correlation-based electric expression method allowing the intuitive visualization of patterns.

*Bombyx mori* (L.) (Lepidoptera: Bombycidae), apart from its economic and agricultural importance, is perhaps the best model species especially for biochemical, molecular genetics and genomic studies in the Lepidoptera (Goldsmith et al. 2005). Previous experimental techniques in molecular biology such as EST sequencing (Mita et al. 2003, 2004; Xia et al. 2004), DNA microarrays (Xia et al. 2007), and serial analysis of gene expression SAGE (Funaguma et al. 2007, Huang et al. 2007, Zhang et al. 2007) produced a large quantity of data. Especially EST sequences, as the main resource, have been widely used in all aspects of genomic research, including the analysis of gene expression patterns. Their growth rate has been startling. Up to 01 July, 2009, the records for EST and UniGene of *B. mori* published on NCBI had reached totals of 245,761 and 11,359, respectively. These resources have been used widely in gene cloning and identification, in the analysis of gene sequence diversity and single nucleotide polymorphism (SNP) research, attracting the attention of many laboratories (Li et al. 2009; Gui et al. 2008; Hong et al. 2006; Yamamoto et al. 2005). The dbEST resource will be used constantly with the emergence of new bioinformatics methods.

SAGEmap is an analysis tool for gene expression profiles developed by NCBI, based on the public SAGE database (Lash et al. 2000). The information obtained from *B. mori* was of low quantity (http://www.ncbi.nlm.nih.gov/sites/entrez) and generally not suitable for gene expression analysis. Microarray-based gene expression profiling on SilkDB (BmMDB, http://www.silkdb.org/microarray/) was per-formed through BLAST sequencing of query genes (Xia et al. 2007). However, this provided information only about 10 tissue types at one development stage. Furthermore, only a few studies have been aware of the importance of comprehensive application of these electronic modes for analyzing gene expression. In the present study, a spatio-temporal expression analysis platform of *B. mori* was constructed with the aid of apEST, with the aim of integrating physiological knowledge into the electric expression profile. Moreover, the platform was also set for assigning protein annotation and GO category (Gene Ontology Consortium 2001), with the aim of expanding new applications of ESTs.

## Materials and Methods

### Theoretical foundation

The frequency of a unique EST (gene) within each stage from cDNA libraries could be determined and could provide a hint for the expression level of that specific gene. The total number of ESTs and the tissues from which they originated are displayed in the cluster browser. The tissues are listed under expression information, which includes the tissue source of libraries of the component sequences (Ewing et al. 1999). UniGene is a system for partitioning GenBank sequences, including ESTs, into a nonredundant set of gene-oriented clusters. Each UniGene cluster contains sequences each representing a unique gene, and is linked to the tissue types in which the gene is expressed. Each UniGene entry is a set of transcript sequences that appear to come from the same transcription locus (gene or expressed pseudogene), together with information on protein similarities, gene expression, cDNA clone reagents and genomic location (Guo et al. 2008).

For the silkworm, up to 01 July 2009, over 245,761 ESTs in GenBank were assigned to 12,081 UniGene clusters. When sufficient genomic sequence is available, UniGene clusters are built using a genome-based clustering system to identify sets of transcript sequences, which correspond to distinct transcription loci or to annotated genes. ESTs were assembled into UniGene clusters that were mapped back to the species using BLAST (http://blast.ncbi.nlm.nih.gov/Blast.cgi), and the level of gene expression was inferred from the number of ESTs in each cluster (Lanier et al. 2008). EST profiles show approximate gene expression patterns as inferred from EST counts and the cDNA library sources.

### Dataset acquisition

In the present study, the publicly available EST database (dbEST) of *B. mori* was analyzed. Using “domestic silkworm” as a key term on NCBI, 245,761 ESTs (up to 01 July 2009) were retrieved and downloaded in FASTA format.

### Dataset processing

apEST was developed (http://jysw.suda.edu.cn/bombyx/Download.html) to run under multiple operating systems using the Java programming language (version 6.0 and later). Using the apEST analysis platform, tissue type, development stage, sex and strain fractions were extracted from dbEST and then classified and summed. Subsequently, corresponding distribution graphs based on EST counts were constructed. On this basis, distributions of information for one tissue at different development stages or at a single development stage for different tissues could also be attained.

### Application and verification of apEST.jar

To demonstrate the apEST platform, a FASTA text that contained the total ESTs ID of one gene was filed by searching the corresponding UniGene information. Then the text file was entered to apEST.jar, and a raw file including information on the expression of the query gene was returned, the numbers of ESTs of every tissue type at the same developmental stage were summed, and the number was converted to transcripts per million (TPM) (TPM = 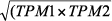 , where TPM 1 and TPM 2 represent one million times the number of ESTs of the query gene divided by the total number of ESTs in each tissue and developmental stage pool, respectively). A spatio-temporal expression profile of this gene was then presented as a three-dimensional columnar graph.

**Figure 1.**
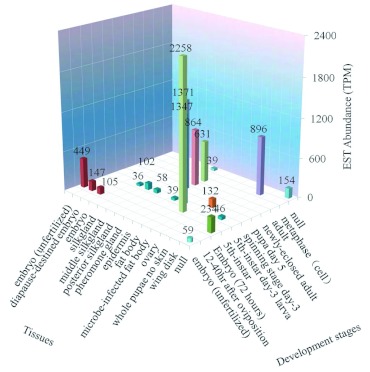
Gene spatio-temporal expression profile of *catalase* was performed by the apEST platform of *Bombyx mori*. EST counts of every tissue type at the same development stage in cat_EST_analysis.txt running by apEST.jar were converted into EST abundance (TPM). High quality figures are available online.

To estimate the apEST analysis platform and contrast with other methods, tissue-prevalent and tissue- and development stage-specific genes were selected by running different platforms. The results were compared with other online analyses of expression profiles, and the results were then matched with these genes' features. This ultimately allowed an objective evaluation of this platform. Then, 37 genes reported from microarray data and RT-PCR results were selected to test the reliability of the apEST platform. The results were finally compared with the results from BmMDB and from actual experiments.

### Protein annotation and GO category

The annotated protein information for invertebrates was downloaded from the UniProt FTP website (ftp://ftp.uniprot.org/pub/databases/uniprot), and dbEST sequences of *B. mori* were also analyzed using BLASTx against the dataset (the e-value cutoff was 1.0 e^-10^). By applying “blast_EST.jar”, a batch protein annotation of the corresponding ESTs was carried out. The most meaningful match was selected and used to compile outputs for each subject to GO categories. Using the Web Gene Ontology Annotation Plot (WEGO) application (http://wego.genomics.org.cn/cgibin/wego/index.pl) (Ye et al. 2006), GO categories for one to three datasets were then shown in a unified plot (Figure 3).

**Figure 2.**
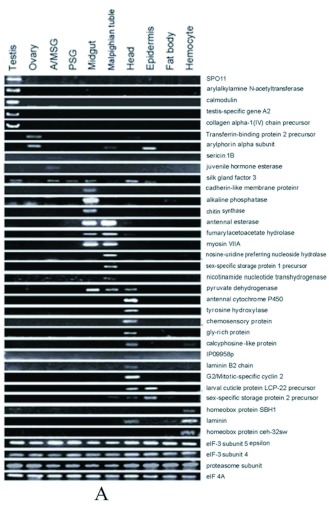
37 genes reported from microarray data (BmMDB) and RT-PCR results were selected to test the reliability of the apEST platform of *Bombyx mori*. A) Expression profile reported from RT-PCR; B) Expression profile run by apEST; The black, red, and white colors indicate genes that are expressed at high, middle, or low levels, respectively. Genes are aligned horizontally, and the tissues are shown vertically. C) Expression profile reported from microarray data. The red, black and green colors indicate genes that are expressed at high, middle, or low levels, respectively. Genes are aligned horizontally, and the tissues are shown vertically. Four genes encoding elF-3 subunit 4, elF 3A subunit 5, elF 4A and proteasome subunit were negative controls. High quality figures are available online.

**Continued.**
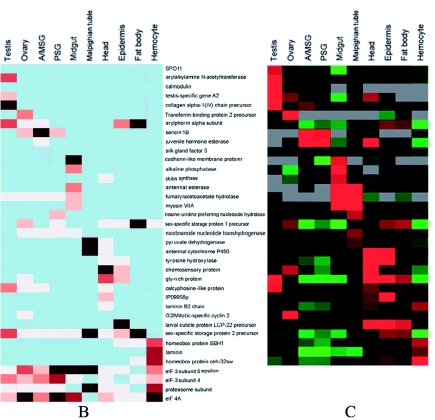

## Results

### Dataset acquisition and classification

From dbEST of *B. mori* downloaded from NCBI, an EST dataset containing information on tissue type, developmental stage, sex and strain was extracted and listed in a text document as an “EST Source.txt”, where one EST was placed on one line and each fraction was separated by a space.

Classification statistics were then compiled. First, a total of 28 tissue types (for convenience of description, every tissue/organ type was considered a tissue) were grouped individually. Of these, the ovary, silk gland, and wing disk showed high EST counts, accounting for 14.9%, 12.4% and 14.6% of the total EST dataset, respectively. This suggests that these tissues were more frequently studied by researchers. The silk gland is a site of highly efficient protein synthesis tightly linked to silk production. The ovary is directly related to reproduction and plays an important role in cell research and genomic studies. Wing disk is good material for exploring regression mechanisms, even for pest control.

Second, a total of 31 developmental stages were gathered. Among these, 5th-instar day 3 larva, a mixture of 5th-instar larva to spinning stage, and 4th-instar larva on day 2 showed high EST counts of 31.2%, 16.8%, and 9.2%, respectively. It is well known that the 5th-instar day 3 larva is at the boundary of larval development, when silk gland cells proliferate and enlarge rapidly and silk protein synthesis commences. Most biological processes are similar during successive feeding stages at and before this time point (Xia et al. 2007). Thus, the study of this time point and other close phases would be helpful to elucidate the regulatory mechanism of the mass synthesis of silk proteins and growth of the silkworm. Third, the Dazao strain (P50) accounted for 85.3% of ESTs from 10 strains. Mixed sex exhibited a very high degree of proportion (49.3%) among four sex types. All these EST counts displayed the popular strains and sex types used in silkworm studies.

**Figure 3.**
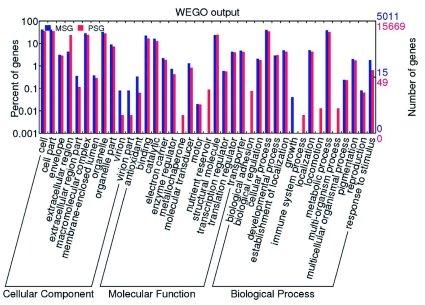
GO categories of genes in middle silk gland (MSG) and posterior silk gland (PSG) of *Bombyx mori*. Although few genes were identified between MSG and PSG, the differences between the two sets of genes were small on the basis of GO annotation. High quality figures are available online.

The constructed platform was also applied to screen spatio-temporal expression information for tissue types and developmental stages of every EST presented simultaneously. Taking the developmental stage of the 5th-instar day 3 larva for example, the tissue distribution at this stage showed that the silk gland and fat body had the highest proportion of ESTs. Studying the silk gland at this phase will be beneficial to learning more about the regulation of fibroin secretion and to clarify the mechanism of high-yield cocoons. Similar distributions for other developmental stages or tissue types can also be attained, which will help in further understanding the regulation of development.

### Application of apEST.jar

As a representative gene, all EST ID of *catalase* (UniGene: Brno. 1023) were listed in one file. After running on apEST.jar, “cat_EST_extraction.txt” and “cat_EST_ analysis.txt” were exported as results files. The main statistical results for the expression of *catalase* are shown graphically based on TPM (Figure1). The temporal-spatial expression profile indicated that the highest expression level of this gene occurred in the microbe-infected fat body at the 5th-instar stage, followed by the fat body of the 5th-instar day 3 larva and the pheromone gland of the day 5 pupa.

Otherwise, *Catalase* expressed in other tissues was absent from apEST.jar, such as malpighian tubule and head from BmMDB platform, for different items among platforms. So after combining them, there were 17 tissue types presented by two pathways together, taking on a comprehensive expression pattern of *catalase*. Confirmed with the results reported from experiments (Yamamoto et al. 2005), it further affirmed that the fat body is the site of highest gene expression and can be taken as the suitable study materials for *catalase*. Moreover, the results by apEST.jar showed that the EST abundance for *catalase* was higher in the microbe-infected fat body. This may be related to the detoxification of the fat body. This indicates that there are opportunities to find some key details on apEST.

**Table 1.**
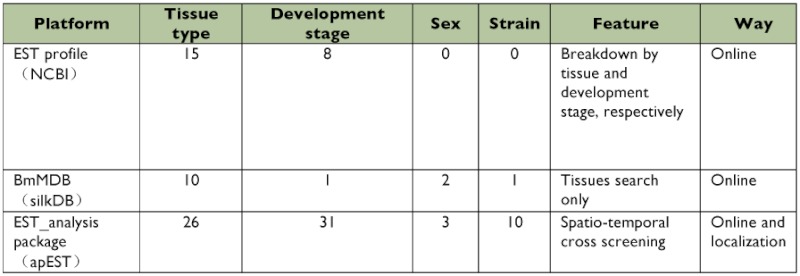
Comparison of three gene expression analysis platforms of *Bombyx mori*.

### Estimation and verification of apEST.jar

The apEST platform has unique items and overlaps with two other analysis approaches: the BmMDB and EST profile online databases (Table 1). The apEST platform employed more items and elucidated the exact expression information for the investigated gene, giving a clearer understanding of the study target. Estimated results for representative genes from the three platforms are shown in Table 2. Running ribosomal protein S2e, encoded by a housekeeping gene (UniGene: Brno.39) of *B. mori*, the results from the three approaches gave similar results with expression in almost all supplied tissue types. These results were consistent with the expression nature of the housekeeping gene. By contrast, serine 3, encoded by a tissuespecific gene (UniGene: Brno.9607) of *B. mori*, was expressed exclusively in the middle silk gland (MSG). This was mirrored by BmMDB and apEST, but could not be identified by the online EST profile database without classification of the functional parts of the silk gland. The same situation occurred in 52 other tissue-specific genes expressed in the MSG and posterior silk gland (PSG) regions. These genes were reported by Xia et al. (2007). *Chorion protein* (UniGene: Brno. 8132), a development stage-specific gene of *B. mori* only expressed in the pupa, could be identified from apEST and the EST profile online database, but could not be identified by BmMDB for any other stage involved except the 5th-instar day 3 larva. To summarize, every method laid particular emphasis on different aspects, but only by combining these three approaches could relatively comprehensive results be obtained.

**Table 2.**
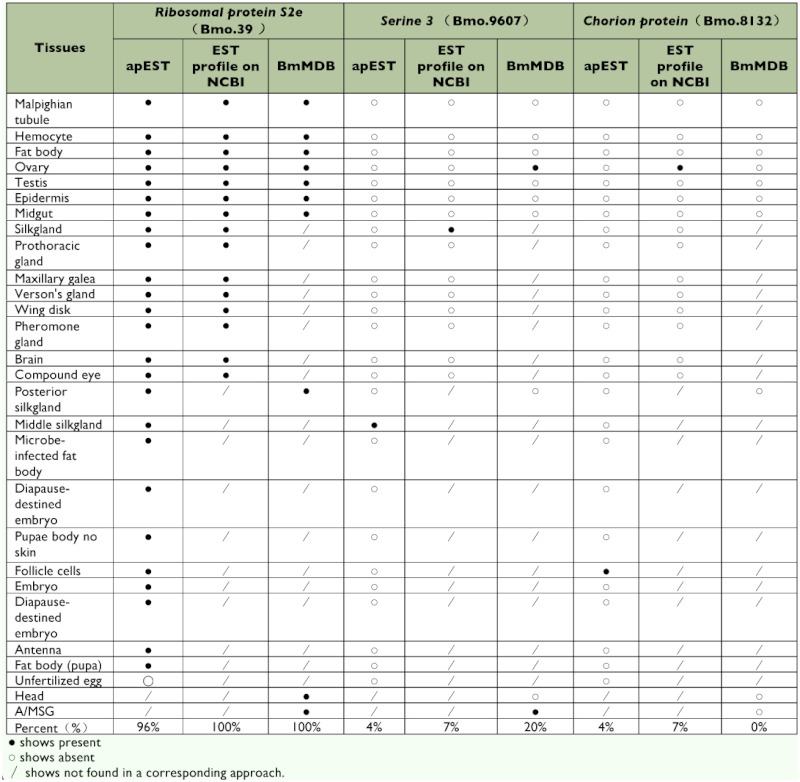
Expression patterns of tissue-prevalent, and tissue- and development stage-specific genes provided by three platforms *of Bombyx mori*.

A total of 37 query genes confirmed from microarray data and RT-PCR experiments were selected running on the apEST.jar. The results had great conformity among platforms (Figure 2). However, there were some special cases. First, *calmodulin* could not be found on UniGene based on probe ID, so the gene expression profile based on EST counts was absent. Secondly, six ESTs belonging to UniGene Bmo.755 (*Silk gland factor 3, POU domain protein Ml*) and distributed in prothoracic gland and pheromone gland, were not presented in one of 10 tissue provided from microarray data and RT-PCR experiment. Thirdly, the UniGene of *Chitin synthase* (Brno. 1774) had only one EST residing in the head, which did not harmonize with the results of RT-PCR in the midgut. Last, the gene of *G2/Mitotic-specific cyclin 2*, highly expressed in the ovary, was consistent with microarray data, but did not harmonize with the result of RT-PCR in the head. Besides these, UniGene IDs of another 2 genes, *myosin VIIA* and *homeobox protein ceh-32*, could not be found. However, based on ESTs linked to their probes, expression profiles using apEST were in harmony with other two results. Different methods had unique features, allowing some differences between them, and by integrating their advantages a more complete gene expression pattern emerged.

### Protein Annotation and GO categories

Operation of blast_EST.jar led to the acquisition of corresponding protein annotations of ESTs and eventually their GO function. The results could be presented by statistical information, protein annotation, and GO category annotations. The EST dataset of MSG and PSG were selected to conduct the process. Protein annotation of the two datasets showed the main features of these two parts of the silk gland clearly; that is, sericin genes were mainly expressed in the MSG, while fibroin genes were mainly expressed in PSG. GO results showed that the two datasets were highly similar (Figure 3). These results indicate that the two tissues might have similar biological functions or be involved in similar physiological processes. By contrast, antioxidants under molecular function was higher in the MSG than in the PSG, which coincides with the antioxidant function of sericin in the MSG. Gene percentages of virion and virion parts were higher in the MSG than in the PSG, suggesting that recombinant viral expression was greater in the MSG than in the PSG. There were some other differences; for example, genes for metallochaperone, nutrient reservoirs, and so on only existed in PSG, but genes for growth only existed in the MSG. clearly this warrants further research.

## Discussion

In summary, a general framework for electronic expression profiling based on the *B. mori* dbEST is proposed. This may have a solid future not only in practice but also in methodology. ESTs and UniGene data of NCBI have a standard format, which makes it possible to extract information from the two documents and provide sufficient information necessary to construct electronic expression profiles. However, it is known that systematic biases in various methods for estimating gene expression levels cannot be ruled out (Munoz et al. 2004). As far as electronic expression profile based on apEST is concerned, there are two possible explanations for biases. On the one hand, ESTs are from cDNA clones randomly. Accordingly, accuracy of gene expression depends on the sequencing coverage. Even though EST counts were converted to TPM, EST profiles show approximate gene expression patterns on the base of reasoning value. On the other hand, some of the EST fractions were submitted by different institutions without unified items, and there is much missing information and unclear parts, such as tissue types of “whole body,” “uncharacterized tissue,” and so on. In addition, up to now, UniGene of *B. mori* reached 12,028 records while microarray data of *B. mori* had 22,987 probes (Xia et al. 2007), so EST profiles are not abundant.

The ultimate aim of bioinformatics is to provide a clue to solve biological problems rather than simply mining a new algorithm. There is now great interest in data analysis and in the extraction of biological significance. In the current study, gene expression was analyzed from dbEST of *B. mori* and physiological knowledge was incorporated into an electronic expression profile. This constructed apEST platform provides systemic investigation and a favorable reference for the excavation of specific expression genes and selection of experiment material. The apEST was tested reliably on current research results and has been carried out in a wide range of applications in the laboratory (Ji et al. 2009). The source data of apEST can be updated easily, as the dbEST of *B. mori* has increased only slowly and has reached saturation. The platform can be also applied to other species by modifying some parameters and can serve as a model for the general study of gene expression in the Lepidoptera.

several online analysis platforms were compared. BmMDB only offers a prediction of expression information for one developmental time point and 10 tissue types, while the online EST profile database cannot screen for expression information on tissue types and development stages simultaneously. Moreover, it is not applicable to some tissue-and stage-specific genes as there is no classification of some tissues and stages, such as the MSG and PSG. Although the apEST platform compensated for these limitations, its sensitivity for those genes with few ESTs submitted was worse than BmMDB. Therefore, integration and complementation of these three approaches was proposed and implemented in this study.

Gene expression quantifying techniques promise to shape understanding of the distribution and regulation of the products of transcription in normal and abnormal cell types. In medicine, the construction and application of electronic gene expression profile from a variety of experimental data, especially between the normal and pathological conditions, has become an important approach to search for mechanisms of many diseases and to assist in disease prevention and treatment (Lü et al. 2007; Li et al. 2004; Su et al. 2003; Gutmann et al. 2002). In the current platform, the microbe-infected fat body presented with up-regulated expression of detoxification enzyme gene, *catalase*. There are other abnormal tissue types, such as BmNPV-infected ovary cells and HCl treated eggs. These will become good material for measuring gene-specific expression and providing evidence to find the genetic mechanisms underlying abnormal states. The candidate genes of interest derived from this dataset will help in comparing the roles of gene expression, function, and evolution using cross-species databases between Lepidoptera and other animals (Chen et al. 2006).

### Additional data files

The following additional data files are available for this manuscript at http://jysw.suda.edu.cn/bombyx/Download.html. Additional data file 1 shows the distribution profiles of tissue type (A), development stage (B), strain and sex (C) based on EST counts from dbEST of Bombyx mori. Additional data file 2 shows tissue distribution profile based on EST counts of 5th-instar day-3 larva of Bombyx mori. Additional data file 3 lists the EST counts in 10 tissues of 37 genes of Bombyx mori. Additional data file 4 lists the protein annotation of ESTs with higher frequency in MSG and PSG.

## Abbreviations

EST: Expressed Sequence Tag;
apEST: EST analysis package;
dbEST: database EST;
GO: Gene Ontology;
TPM: transcripts per million;
BmMDB: Microarray-based gene expression profile on SilkDB;
MSG: middle silkgland;
PSG: posterior silkgland
